# Systematic strategies for developing phage resistant *Escherichia coli* strains

**DOI:** 10.1038/s41467-022-31934-9

**Published:** 2022-08-02

**Authors:** Xuan Zou, Xiaohong Xiao, Ziran Mo, Yashi Ge, Xing Jiang, Ruolin Huang, Mengxue Li, Zixin Deng, Shi Chen, Lianrong Wang, Sang Yup Lee

**Affiliations:** 1 Department of Gastroenterology, Ministry of Education Key Laboratory of Combinatorial Biosynthesis and Drug Discovery, Zhongnan Hospital of Wuhan University, School of Pharmaceutical Sciences, Wuhan University, Wuhan, Hubei 430071 China; 2 Department of Burn and Plastic Surgery, Shenzhen Institute of Translational Medicine, Health Science Center, Shenzhen Second People’s Hospital, the First Affiliated Hospital of Shenzhen University, Shenzhen, Guangdong 518035 China; 3 Department of Chemical and Biomolecular Engineering (BK21 Four Program), Korea Advanced Institute of Science and Technology, Yuseong-gu, Daejeon, 34141 Republic of Korea

**Keywords:** Applied microbiology, Bacteriophages, Metabolic engineering

## Abstract

Phages are regarded as powerful antagonists of bacteria, especially in industrial fermentation processes involving bacteria. While bacteria have developed various defense mechanisms, most of which are effective against a narrow range of phages and consequently exert limited protection from phage infection. Here, we report a strategy for developing phage-resistant *Escherichia coli* strains through the simultaneous genomic integration of a DNA phosphorothioation-based Ssp defense module and mutations of components essential for the phage life cycle. The engineered *E. coli* strains show strong resistance against diverse phages tested without affecting cell growth. Additionally, the resultant engineered phage-resistant strains maintain the capabilities of producing example recombinant proteins, D-amino acid oxidase and coronavirus-encoded nonstructural protein nsp8, even under high levels of phage cocktail challenge. The strategy reported here will be useful for developing engineered *E. coli* strains with improved phage resistance for various industrial fermentation processes for producing recombinant proteins and chemicals of interest.

## Abstract (web summary)

Phage contamination is a persistent problem in industrial biotechnology processes employing bacterial strains. Here, the authors report the construction of *E. coli* host strains with broad antiphase activities via the genomic integration of the Ssp defense system and mutations of components essential for phage infection cycles.

## Introduction

Constituting the most predominant biological organisms on Earth, bacteriophages or phages are obligate parasites that multiply inside bacteria by hijacking the host biosynthetic machinery and releasing phage progeny to infect neighboring cells^1,2^. To proliferate in phage-rich environments, bacteria have developed defense systems involving diverse mechanisms targeting different steps of phage infection cycles^3,4^. These antiphage mechanisms include inhibition of phage adsorption^5^, blocking the entry of phage DNA^6^, limiting phage growth^7^, abortive infection^8^, restriction-modification (R-M)^9^, toxin-antitoxin^10^, and CRISPR/Cas systems^11^, as well as the recently discovered BREX^12^, DISARM^13^ and Dnd^14^ defense systems. However, the constant battle for survival drives phages to evolve various counterstrategies to overcome these antiphage defense systems^15^. The ongoing evolutionary phage-host arms race has far-reaching impacts on global nutrient cycling^16^, food and biotechnology industries^17^, and human health and disease^18^.

In addition to being an issue in laboratories, phage contamination is a persistent problem in industrial biotechnology processes employing bacterial strains. Phage contamination results in fermentation failure, low-quality and inconsistent end-products, and consequently considerable economic loss^19^. Phage infection incidents have occurred in various industrial processes worldwide involving the fermentation of milk^20^, cheese^21^ or cucumber^22^ by lactic acid bacteria (LAB), 1,3-propanediol by *Klebsiella pneumoniae*^23^, acetone-butanol-ethanol by *Clostridium saccharoperbutylacetonicum*^24^, and other products. Since the first description of phage contamination in the dairy industry in 1935, an array of approaches have been employed to reduce the risks of phage-induced fermentation failures by different industries, including raw material treatments, starter strain rotation, process changes, adapted factory design, and extensive cleaning and sanitation^19,25^. Moreover, the introduction of plasmid-encoded natural antiphage strategies, e.g., CRISPR-Cas^26^, Abi^27^, antisense mRNA^28^ and R-M systems^27,29^, into starter strains has been reasonably successful, mainly in the dairy industry, to render the strains phage resistant.

We recently identified SspABCD-SspE as a type of bacterial defense system with functions analogous to those of DNA methylation-based R-M systems^30^. The four-gene product SspABCD catalyzes DNA phosphorothioate (PT) modification within a 3-bp 5ʹ-CCA-3ʹ consensus sequence, in which the nonbridging oxygen in the sugar-phosphate backbone between two dCs is replaced by sulfur, yielding the PT-modified product 5ʹ-C_PS_CA-3ʹ (PS: phosphate sulfur linkage). SspE functions as the restriction counterpart that uses 5ʹ-C_PS_CA-3ʹ as the recognition tag to accomplish self vs. nonself discrimination and attacks non-PT-modified invasive phage DNA^30^. Notably, SspE depends on PT modification at 5ʹ-C_PS_CA-3ʹ in the host genome to exert its antiphage activity, which renders Ssp a PT-modulated defense system^30^. Generally, host killing resulting from restriction endonucleases (REases) in R-M systems occurs if the DNA methylation status is disrupted^31^. Moreover, once established in a host cell, the loss of R-M gene complexes can lead to cell death through restriction breakage in the genome, a process known as postsegregational cell killing^32^. In contrast, SspE exerts no toxicity on host cells lacking *sspABCD*, differing from the activity of REases in methylation-based R-M systems or DndFGH in PT-based Dnd systems^33^. This makes the Ssp defense system valuable in providing host protection against phage infection without risking cell viability.

In this work, we choose the *E. coli* MG1655, MG1655 (DE3), and W3110 strains, which are widely used in laboratory and industrial bioprocesses, with the objective of constructing *E. coli* host strains with broad antiphage activities through the genomic integration of the Ssp defense system and mutations of components essential for phage infection cycles. The resulting three engineered *E. coli* strains, MG1655-EPR, W3110-EPR and MG1655 (DE3)-EPR, exhibit growth dynamics that are the same as those of their parental strains and maintain the capabilities of producing example recombinant proteins, D-amino acid oxidase and coronavirus-encoded nonstructural protein nsp8, even in the presence of high-titer phage lysates and a phage cocktail, showing considerable potential for applications in industrial fermentation processes.

## Results

### Validation of SspBCDE defense in commonly used laboratory and industrial *E. coli* strains

**Fig. 1:**
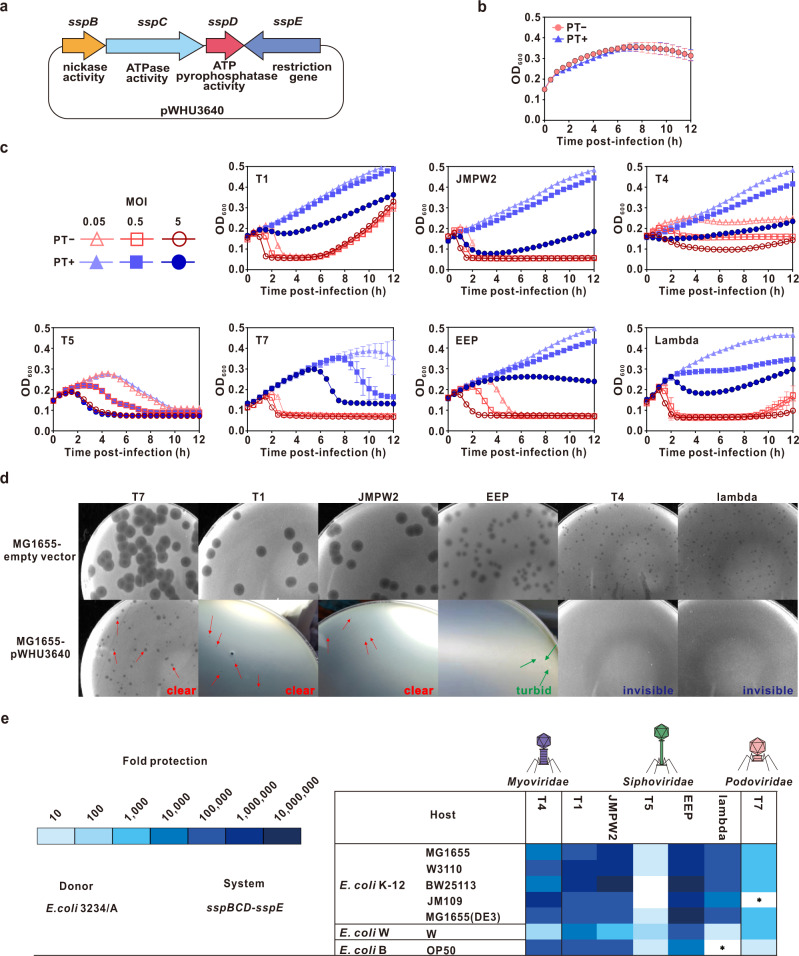
The SspBCDE defense system provides wide-ranging protection against coliphages in industrial *E. coli* strains. **a** Plasmid pWHU3640 containing the *sspBCDE* module from *E. coli* 3234/A is schematically shown. **b** Expression of the SspBCDE defense system in *E. coli* MG1655 does not impair cell growth. Data are shown as the mean ± SD of three independent experiments. **c** The SspBCDE defense system provides protection against coliphages T1, JMPW2, T4, T5, T7, EEP, and lambda in *E. coli* MG1655. Bacteria were infected at *t* = 0 at multiplicities of infection (MOIs) of 5, 0.5 and 0.05. LB was used as a reference, and a final concentration of 100 µg/mL ampicillin was added to the medium to maintain the control empty vector and plasmid pWHU3640. Data are shown as the mean ± SD of three independent experiments. **d** Plaque morphologies of T1, JMPW2, T4, T7, EEP and lambda on *E. coli* MG1655 (pWHU3640) expressing SspBCDE. The red arrows indicate clear plaques of the phages, and the green arrows indicate turbid plaques of the phages. Experiments were repeated at least three times with similar results. **e** The SspBCDE defense system provides protection against coliphages in different *E*. coli hosts. Fold protection was determined by comparing the efficiency of plating (EOP) of phages on the pWHU3640-containing strain to the EOP on a strain that contains an empty vector. Data are shown as the mean ± SD of three independent experiments. * indicates that the coliphage did not infect this host. Source data are provided as a Source Data file.

To evaluate the performance of the PT-based Ssp defense system in *E. coli* strains that are widely used in laboratory and industrial bioprocesses, *E. coli* K-12 MG1655, which lacks endogenous *ssp* genes, was transformed with pWHU3640, harboring the *sspBCDE* gene cassette from *E. coli* 3234/A (Fig. 1a). The *sspA* gene is not clustered with *sspBCDE* in *E. coli* 3234/A, consistent with the fact that the cysteine desulfurase function of SspA can be performed by an IscS ortholog located elsewhere in the genome^34^. The expression of plasmid-encoded genes for SspBCDE conferred DNA PT modification and led to no change in growth dynamics in *E. coli* MG1655 (pWHU3640) compared with those of the control strain transformed with the empty vector pBluescript II SK(+) (Fig. 1b and Supplementary Fig. 1). Then the *E. coli* MG1655 (pWHU3640) cells were challenged with lytic coliphages from all three morphological families of the order *Caudovirales*, including the siphophages T1, JMPW2, T5, lambda and EEP; the myophage T4; and the podophage T7. Infection experiments were performed at multiplicities of infection (MOIs) of 0.05, 0.5 and 5. The SspBCDE defense system introduced into *E. coli* MG1655 provided various degrees of protection against all phages assayed (except for T5), which could be seen by the absence or delay of cell culture collapse upon infection with phages (Fig. 1c). Additionally, introduction of the SspBCDE system resulted in a significant reduction in plaque size and changes in plaque morphology from clear to turbid or almost invisible (Fig. 1d). To quantify the protection against phages, the phage efficiency of plating (EOP) on *sspBCDE*-expressing MG1655 (pWHU3640) relative to that on *ssp*-lacking MG1655 (SK+) was measured. The SspBCDE system conferred strong protection against most of the phages, providing up to six orders of magnitude of protection (Fig. 1e and Supplementary Data 1). The EOP data confirmed that phage T5 was insensitive to SspBCDE defense, which is believed to be associated with the unusual two-step injection of T5 DNA into the host cell^35^. Moreover, the plasmid-borne *sspBCDE* module also gave strong phage resistance in other laboratory and industrial *E. coli* strains, including *E. coli* K-12 strains MG1655 (DE3), W3110, BW25113 and JM109, *E. coli* B OP50 and wild-type *E. coli* W (Fig. 1e, Supplementary Data 1 and Supplementary Fig. 2). These results suggest that the SspBCDE defense system allows general protection of laboratory and industrial fermentation processes involving a broad range of *E. coli* strains from phage attacks.

### Increasing phage resistance by *E. coli* genome engineering

While the introduction of a plasmid-encoded defense system is an easy and convenient way to confer host cell resistance to phages, the application of this method is sometimes limited because of plasmid incompatibility, instability, copy number viability, and the metabolic burden of plasmid propagation and maintenance^36,37^. For the generation of a stable phage-resistant strain, an 8 kb DNA fragment encoding SspBCDE was integrated into the MG1655 chromosome to produce the MG1655-PT strain by using the pKOV-Kan gene replacement vector and lambda red-mediated homologous recombination^38,39^. Integration proceeded at the safe site 9 (SS9) in the genome, which is an intergenic genomic site offering a high integration efficiency and gene expression level without reducing cell viability^40^. Typically, the ratio of modification and restriction activities in R-M systems is within a narrow window of flexibility to balance protection from invasive phage DNA and unwanted host cell death from autoimmunity. While MG1655-PT adopted the same growth profile as MG1655 (pWHU3640), the quantitative RT–PCR results showed that the expression ratio between *sspE* and *sspBCD* in MG1655-PT was slightly higher than that in MG1655 (pWHU3640) (Supplementary Table 1 and Supplementary Fig. 3). Consequently, the MG1655-PT strain showed at least 10-fold increased protection against phages T1, T4, EEP, and lambda (Supplementary Data 1).

**Fig. 2:**
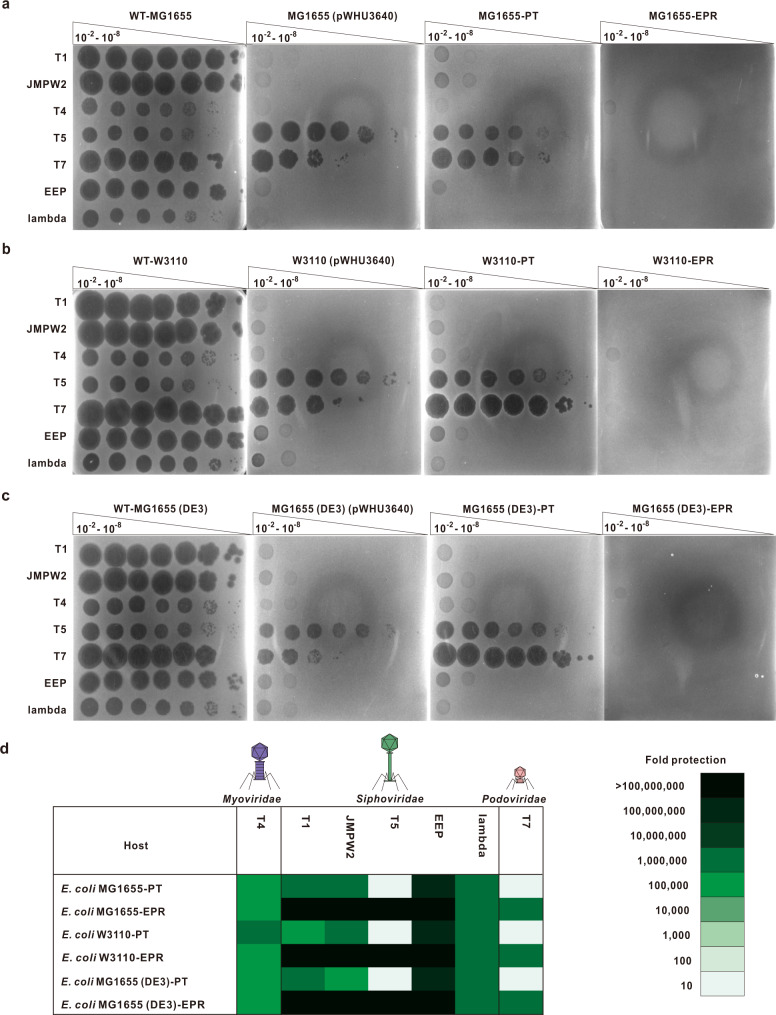
Construction of engineered phage-resistant (EPR) *E. coli* strains. **a**–**c** Phage plaque assays were used to compare the phage infection efficiencies in WT-MG1655, MG1655 carrying pWHU3640, MG1655-PT and MG1655-EPR strains (**a**); in WT-W3110, W3110 carrying pWHU3640, W3110-PT and W3110-EPR strains (**b**); and in WT-MG1655 (DE3), MG1655 (DE3) carrying pWHU3640, MG1655 (DE3)-PT and MG1655 (DE3)-EPR strains (**c**). The results are representative of three independent experiments. **d** The systematic genome engineering strategy confers strong phage resistance on different *E. coli* hosts. EOPs were determined to assess the protection levels of *E. coli*-PT and *E. coli*-EPR strains against coliphages. All the data were obtained at least three times. Source data are provided as a Source Data file.

To achieve a sufficient level of tolerance to phages T5 and T7, the elements in the MG1655-PT strain that are essential to the infection cycle of these phages were modified. T1, T5, and **ϕ**80 recognize FhuA, a ferrichrome transporter of the *E. coli* outer membrane^41^, as the receptor for phage DNA injection, while T7 adopts thioredoxin (TrxA) in the host cell as a processivity factor of T7 DNA polymerase for phage DNA synthesis^42^. The deletion of TrxA and extracellular loop 8 of FhuA in *E. coli* MG1655-PT resulted in the engineered phage-resistant (EPR) strain MG1655-EPR (Fig. 2a and Supplementary Fig. 4a), which exhibited extremely strong resistance against all the coliphages tested, as manifested by plaques barely forming on the lawn of MG1655-EPR cells even at the highest phage titer tested (Fig. 2a). The same genomic engineering strategy of integrating *sspBCDE* and mutating *fhuA* and *trxA* was performed in two additional genetically similar *E. coli* K-12 strains, W3110 and MG1655 (DE3) (Supplementary Fig. 5), to construct the W3110-EPR and MG1655 (DE3)-EPR strains, respectively; these engineered strains also exhibited tolerance to various phages (Fig. 2b–d and Supplementary Fig. 4b, c).

**Fig. 3:**
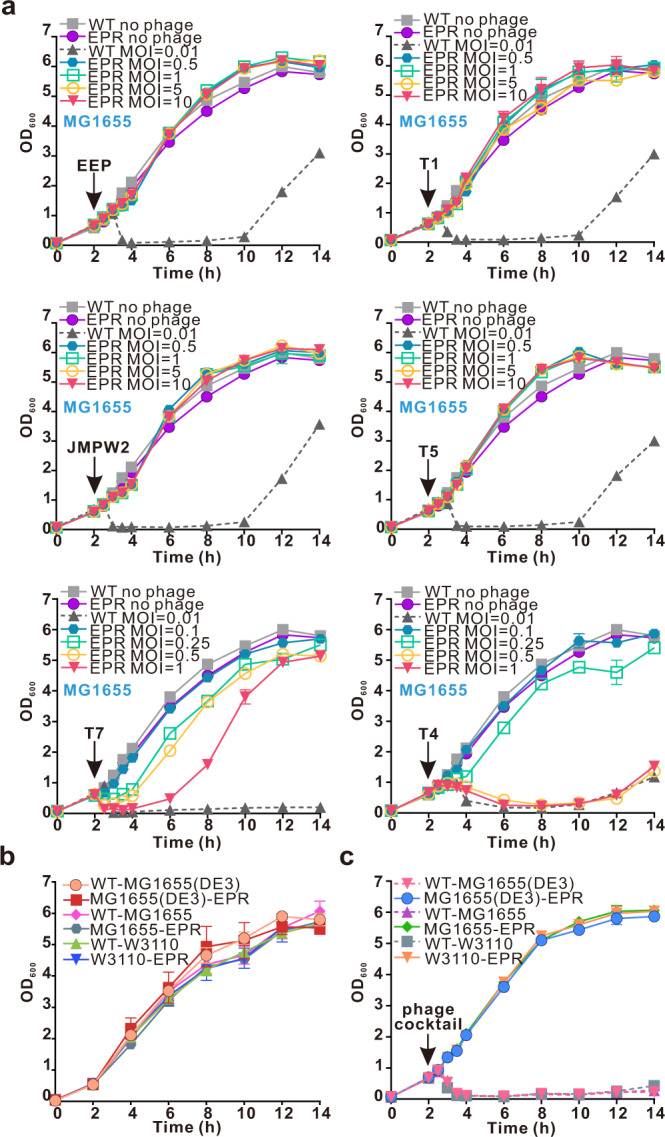
Growth curves of the MG1655-EPR strain treated with coliphages at a range of MOIs. **a** MG1655-EPR strains were treated with the coliphages EEP, T1, T5, JMPW2, T7, and T4 at the indicated MOIs. WT-MG1655 and MG1655-EPR strains not treated with phages and WT-MG1655 treated with phages at MOI = 0.01 were set as control groups. Growth curves of W3110-EPR and MG1655(DE3)-EPR strains treated with coliphages at the indicated MOIs are shown in Supplementary Fig. 6. Experiments were repeated three times, and each point represents the mean ± SD. **b**, **c** Growth curves of wild-type and EPR *E. coli* strains not treated (**b**) or treated (**c**) with phage cocktail. The phage cocktail was composed of T1 (MOI = 10), JMPW2 (MOI = 10), T4 (MOI = 0.1), T5 (MOI = 10), T7 (MOI = 0.1), EEP (MOI = 10), and lambda (MOI = 0.5) and was added when the OD_600_ reached 0.6. Experiments were repeated three times, and each point represents the mean ± SD. Source data are provided as a Source Data file.

Growth curves obtained in the presence of phages T1, T5, EEP, and JMPW2 at varying MOIs showed that no lysis of MG1655-EPR, MG1655 (DE3)-EPR, or W3110-EPR was observed even at the highest MOI of 10 (Fig. 3a and Supplementary Fig. 6). While phages T4, T7 and lambda inhibited *E. coli* growth in an MOI-dependent manner, the three EPR strains showed significant resistance against phages T4 and T7 at an MOI of 0.1 and phage lambda at an MOI of 0.5 (Fig. 3a and Supplementary Fig. 6). Thus, engineering the genome by the integration of *sspBCDE* and mutation of *fhuA* and *trxA* is an effective strategy for producing *E. coli* strains that are highly resistant to a broad range of phages (Supplementary Fig. 7).

### Application of engineered phage-resistant *E. coli* strains in recombinant protein production

**Fig. 4:**
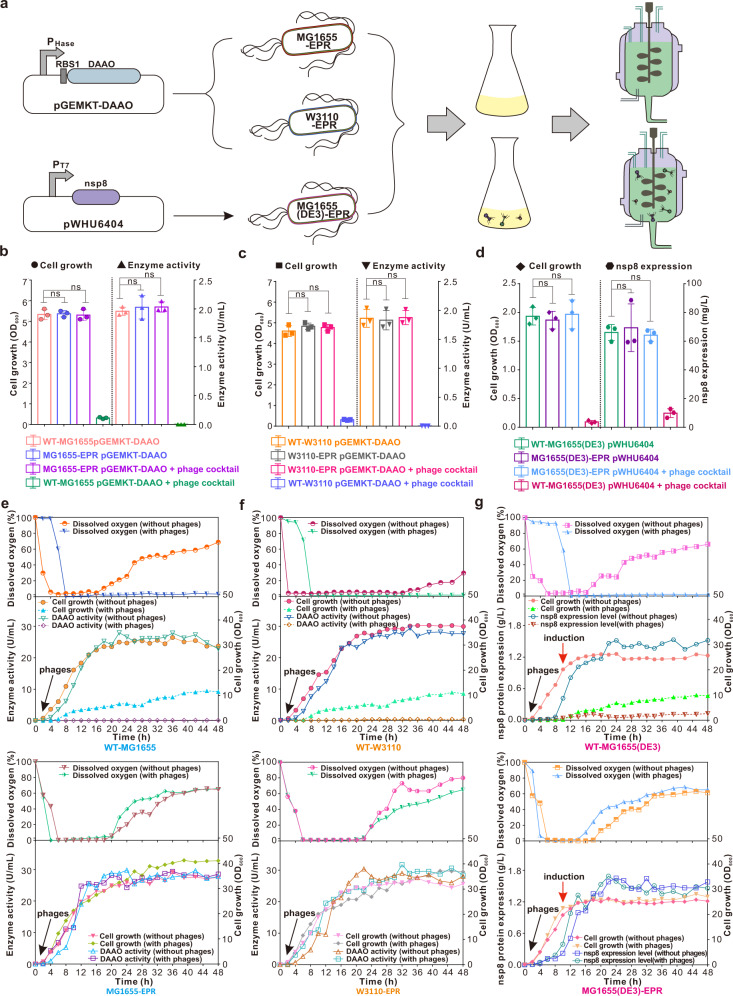
Application of EPR *E. coli* strains in phage-rich flask-shaking or fed-bath recombinant protein production. **a** Flow chart of EPR *E. coli* strains applied in recombinant protein production. **b**-**d** Flask-shaking recombinant protein production characteristics of the wild-type and EPR *E. coli* strains when infected or not infected with phage cocktail. WT-MG1655 and MG1655-EPR strains producing DAAO (**b**), WT-W3110 and W3110-EPR strains producing DAAO (**c**), and WT-MG1655 (DE3) and MG1655 (DE3)-EPR strains producing nsp8 (**d**) are shown. Phage cocktail was added at the start of cultivation. Values represent the mean of three biological replicates, and error bars represent standard deviations (dots). Statistical significance was calculated by one-way ANOVA followed by Tukey’s test. ns, not significant. **e**–**g** Average fed-batch fermentation profile of the wild-type and EPR *E. coli* strains when infected or not infected with phage cocktail. WT-MG1655 and MG1655-EPR strains producing DAAO (**e**), WT-W3110 and W3110-EPR strains producing DAAO (**f**), and WT-MG1655 (DE3) and MG1655 (DE3)-EPR strains producing nsp8 (**g**) are shown. Phage cocktail was added at the start of fermentation (black arrow), and IPTG was added when the OD_600_ reached 20 (red arrow). Values represent the means of duplicate fermentation profiles. Source data are provided as a Source Data file.

The genomic integration of *sspBCDE* and mutations of *fhuA* and *trxA* had no inhibitory effect on cell growth in the resultant EPR *E. coli* strains tested (Fig. 3b). In addition, the EPR *E. coli* strains showed strong resistance against the coliphage cocktail containing phages T1, T4, T5, T7, EEP, JMPW2, and lambda (Fig. 3c). Thus, we decided to examine whether these EPR strains can be employed for the production of recombinant proteins while providing protection against phage infection. As three example systems, *E. coli* MG1655-EPR and W3110-EPR strains producing D-amino acid oxidase (DAAO) and *E. coli* MG1655 (DE3)-EPR strains producing a nonstructural protein of coronavirus SARS-CoV-2 (nsp8) were constructed and tested (Fig. 4a). DAAO catalyzes the oxidative deamination of D-amino acids in a flavin adenine dinucleotide (FAD)-dependent manner and has been widely used as an industrial biocatalyst to produce enantiomerically pure amino acids and convert cephalosporin C (CPC) to 7-aminocephalosporanic acid^43^. The nps8 protein is a key component of the SARS-CoV-2 RNA-dependent polymerase complex, a promising antiviral target^44^.

The plasmid pGEMKT-DAAO^45^, which constitutively expresses the DAAO-encoding *dao* gene from *Trigonopsis variabilis*, was transformed into MG1655-EPR and W3110-EPR and examined for DAAO enzyme activity in 2 L shake flasks containing 1 L of lysogeny broth (LB). When inoculated together with a phage cocktail consisting of 4.0 × 10^9^ PFU/L each of T4 and T7, 2.0 × 10^10^ PFU/L of lambda and 4.0 × 10^11^ PFU/L each of T1, JMPW2, T5, and EEP, the wild-type MG1655 and W3110 cells harboring pGEMKT-DAAO rapidly died, whereas MG1655-EPR and W3110-EPR harboring pGEMKT-DAAO still showed robust growth (Fig. 4b, c). The MG1655-EPR and W3110-EPR strains harboring pGEMKT-DAAO increased to OD_600_ values of ~5.3 and 4.8, respectively, after 12 h of cultivation at 37 °C, which were comparable to those obtained in the absence of phages. While showing strong tolerance to phages, MG1655-EPR (pGEMKT-DAAO) and W3110-EPR (pGEMKT-DAAO) produced DAAO with activities of 2.03 ± 0.07 and 1.88 ± 0.10 U/mL, respectively, using D-alanine as a substrate. These values are comparable to those obtained with the parent MG1655 and W3110 strains harboring pGEMKT-DAAO without phage challenge (Fig. 4b, c).

To further investigate the applicability of the proposed antiphage genome engineering strategy, the broadly employed T7 expression system was examined next. To express nsp8, the encoding gene was cloned into the pET-28a(+) vector under the control of the T7 RNA polymerase promoter to construct pWHU6404. The MG1655 (DE3)-EPR strain harboring pWHU6404 was cultured at 37 °C in 1 L of LB medium. When the OD_600_ reached 0.6–0.8, 0.1 mM IPTG was added to induce the expression, and the cells were further grown at 28 °C for 18 h. The majority of the nsp8 protein produced was present in a soluble form, and the concentration of soluble fusion protein produced from MG1655 (DE3)-EPR (pWHU6404) reached ~64 mg/L even when the culture was incubated with phage cocktail, which is comparable to that obtained with the parent MG1655 (DE3) strain harboring pWHU6404 without phage challenge (Fig. 4d). Notably, over the time of 47 transfers (transferred every 12 h) corresponding to approximately 400 generations, all three EPR strains maintained strong phage resistance as well as target protein production, demonstrating their genome and fermentation stability (Supplementary Fig. 8).

### Fed-batch fermentation performance of EPR *E. coli* strains in the presence of phage cocktail

Fed-batch fermentation is the most preferred and frequently used cultivation method for industrial fermentation, requiring a prolonged cultivation period with intermittent or continuous feeding of nutrients and gases (e.g., air or oxygen in aerobic fermentation). Due to these operation characteristics, fed-batch fermentation is more vulnerable than batch fermentation to phage attack. We thus examined whether the EPR *E. coli* strains can tolerate phage attack during fed-batch fermentation. Three engineered strains, MG1655-EPR (pGEMKT-DAAO), W3110-EPR (pGEMKT-DAAO), and MG1655(DE3)-EPR (pWHU6404), together with their counterpart control strains, were cultured in a 5 L fermenter. For the production of DAAO, the cultivation temperature was maintained at 37 °C, and the pH value was adjusted to 7.5 by NaOH. The phage cocktail at a final concentration of 5.0 × 10^9^ PFU/L each of T4 and T7, 2.5 × 10^10^ PFU/L of lambda and 5.0 × 10^11^ PFU/L each of T1, JMPW2, T5, and EEP was added to the fermenter at the start of fermentation. Upon phage infection, the MG1655 and W3110 cells multiplied at a significantly lower rate than the uninfected cells (Fig. 4e, f). In sharp contrast, the two EPR strains MG1655-EPR (pGEMKT-DAAO) and W3110-EPR (pGEMKT-DAAO) displayed growth profiles similar to those of the control strains without phage infection. Without the addition of phage cocktail, MG1655-WT (pGEMKT-DAAO) and MG1655-EPR (pGEMKT-DAAO) produced DAAO with similar activities of 22.81 ± 2.00 and 27.76 ± 0.75 U/mL, respectively, in 48 h. Meanwhile, even in the presence of a high-concentration phage cocktail, MG1655-EPR (pGEMKT-DAAO) also maintained a consistent DAAO activity of 28.53 ± 2.79 U/mL in 48 h, while almost no DAAO activity was detected in the fermentation broth of MG1655-WT (pGEMKT-DAAO) due to the devastating effect of the phage cocktail on the cells (Fig. 4e). Analogously, W3110-WT (pGEMKT-DAAO) without phage cocktail challenge and W3110-EPR (pGEMKT-DAAO) without or with phage cocktail challenge produced DAAO activities of 27.65 ± 2.89, 28.05 ± 0.25 and 28.44 ± 2.27 U/mL in 48 h, respectively, which indicated that the resultant engineered phage-resistant strains maintained the capabilities of producing DAAO while resisting high levels of phage cocktail attack (Fig. 4f). The dissolved oxygen (DO) concentration profiles during the fermentation of these two EPR strains were also similar to those monitored during the fermentation of their respective parent wild-type strains, even under the high-concentration phage cocktail challenge (Fig. 4e, f).

Fed-batch culture of MG1655 (DE3)-EPR (pWHU6404) was conducted in a 5 L fermenter at the initial cultivation temperature of 37 °C to allow cells to grow rapidly. When the OD_600_ reached 20, the temperature was adjusted to 28 °C, and IPTG was added to a final concentration of 1 mM. The high-concentration phage cocktail was also added to the fermenter at the start of fermentation. From the perspective of cell growth, almost the same cell density was reached at the stationary phase in a time range of 16–48 h among the experimental groups of MG1655 (DE3)-WT (pWHU6404) without phage cocktail challenge and MG1655 (DE3)-EPR (pWHU6404) without or with phage cocktail challenge. The nsp8 protein was functionally expressed in a soluble form in all three fermentations, with expression levels of 1.52 ± 0.16, 1.58 ± 0.06 and 1.46 ± 0.09 g/L, respectively (Fig. 4g). These results suggest that engineered MG1655 (DE3)-EPR is a promising *E. coli* host for the large-scale production of heterologous proteins with a high level of phage resistance. Moreover, no obvious genomic changes in EPR *E. coli* strains were detected by ligation-mediated polymerase chain reaction (LM-PCR)^46^ even in the presence of phage cocktail after prolonged incubation time, demonstrating the genomic stability and integrity of the EPR *E. coli* strains in phage-rich environments (Supplementary Fig. 9).

Collectively, the above results suggest that the antiphage genome engineering strategy developed here allows the production of *E. coli* strains that are strongly resistant to a broad range of phages without negatively affecting cell growth or recombinant protein production.

## Discussion

**Fig. 5:**
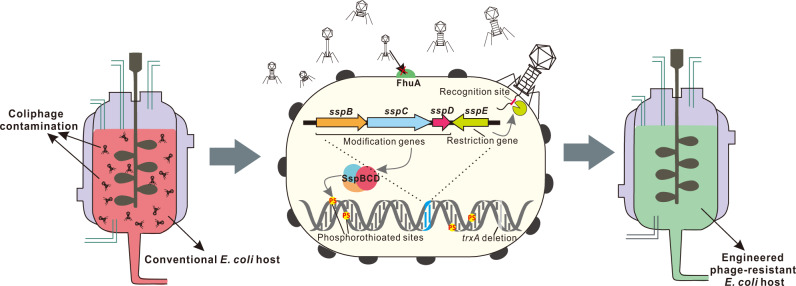
A schematic model of the systematic strategy for engineering phage-sensitive industrial *E. coli* strains into strains with broad antiphage activities. Through the simultaneous genomic integration of a DNA phosphorothioation-based Ssp defense module and mutations of components essential for the phage life cycle, the engineered *E. coli* strains show strong resistance against diverse phages tested and maintain the capabilities of producing example recombinant proteins, even under high levels of phage cocktail challenge.

Bacteriophages constitute one of the greatest threats in industrial bacterial fermentation and can cause fermentation failure and lingering contamination of the fermentation facility. These effects in turn lead to not only a decrease in facility productivity but also enormous financial losses. Thus, it is of prime importance to limit phage entry within manufacturing facilities and prevent phage proliferation in bacterial fermentation processes, which is quite difficult. In this work, we report the construction of engineered laboratory and industrial *E. coli* strains with strong resistance against a wide array of phages through the simultaneous genomic integration of a PT-based Ssp defense module and mutations in the components that are essential to the infection cycles of certain phages (Fig. 5).

Typically, phage resistance mechanisms can be divided into four types: prevention of phage adsorption, blockage of phage DNA injection, inhibition of phage DNA replication, and abortive infection mechanisms^47^. To prevent phage adsorption, bacteria can modify, mask or block phage receptors on the cell surface. However, the application of this defense mechanism provides the host with only a limited effect on a narrow range of phages, typically those that recognize the specific receptor. When the host encounters phages that recognize different receptor sites on the cell surface, this defense mechanism fails^48^. In contrast, R-M- and CRISPR-Cas-mediated inhibition of phage DNA replication has been extensively studied. Generally, R-M systems comprise two contrasting enzymatic activities: a methyltransferase that catalyze the transfer of a methyl group to nucleobases within a consensus motif of ‘self’ DNA and a restriction endonuclease that recognizes and cuts invasive DNA that possesses the same DNA motif when not methylated. As another defense barrier that acts at the nucleic acid level, CRISPR-Cas systems undermine the integrity of invading phage DNA by recognizing and attacking the target sequence, termed the protospacer, in the phage genome that the bacterium has encountered before^49,50^. The CRISPR-Cas defense mechanisms mediated by identifying specific sequence information have a relatively narrow restriction range on bacteriophages, since these target sequences are not present in all phage DNA. Comparatively, the restriction of phages by R-M systems is independent of long sequences; therefore, potentially all phage DNA that was not previously modified will be destroyed by restriction endonucleases.

In *Lactococci*, one of the most important LAB in the dairy industry, many of the natural phage resistance mechanisms, including inhibition of phage adsorption, R-M, CRISPR-Cas and abortive infection systems, are encoded by chromosomal or plasmid DNA^51^. Therefore, the conjugation of native phage-resistant plasmids has been a profitable strategy to genetically improve dairy LAB. The resultant multiple dairy starter cultures have been in commercial use for years. However, it is worth noting that any threat to the maintenance of the intracellular R-M systems can lead to cell death through restriction breakage in the host genome, which is termed postsegregational or post-disturbance cell killing^32^. In contrast, this situation does not occur in the PT-based Ssp system because the restriction component SspE exerts antiphage activity only after being activated by sequence-specific PTs^30^. Moreover, while the conjugal transfer of phage resistance plasmids is one of the simplest and most convenient strategies to improve the phage resistance of LAB starter strains, the isolation of bacteriophage-insensitive mutants (BIMs) is an alternative approach to transform phage-sensitive strains to phage-resistant derivatives by long-term exposure of the phage-sensitive strains to virulent phages^51^. Several studies have described the generation of spontaneous phage-resistant descendants from phage-sensitive LAB strains of *Lactococcus*^52^, *Lactobacillus helveticus*^53^, *Lactobacillus delbrueckii*^54^ and *Streptococcus thermophilus*^55^. The mechanism involved in BIM generation has been found to be associated with CRISPR-Cas systems or abortive infection systems^56,57^. Although this BIM selection approach has the convenience of simplicity and involves no genetic manipulation, it entails some drawbacks, including a high frequency of resistance phenotype reversion, physiological bacterial modifications and altered cell growth^58^.

The ongoing arms race between bacteria and phages has led to the evolution of counterstrategies in phages to evade bacterial defense systems. Phage T5 has been found to be insusceptible to restriction by PT-based Dnd and Ssp systems, and methylation-based *Eco*RI, *Eco*RII, *Eco*P1I and *Eco*K R-M systems^30,35,59^. This resistance is believed to be related to the unusual features of phage T5. Phage T5 differs from most phages because its DNA injection occurs in two steps: the first step transfer (FST) regions, the left-most 7.9% of the T5 genome, are injected and its genes are expressed, and only then does the remainder of the phage DNA (the second step of transfer sequence, SST) enter the host^60^. In addition to facilitating SST entry, the FST region that encodes 16 putative pre-early ORFs is involved in host DNA degradation, inhibition of DNA methylation, and restriction insensitivity^61,62^. Unusually, although T5 DNA contains the recognition sites of *Eco*RI and is sensitive to *Eco*RI cleavage in vitro, T5 is incapable of being modified or restricted within host cells. Although the molecular mechanism of how phage T5 evades the defense systems remains unclear, possibilities have been suggested that T5 DNA carries a DNA protective protein, likely being coded by the FST region, against restriction enzymes or encode an Ocr-like DNA mimic protein to protect T5 DNA not being targeted by R-M systems^63,64^.

Our recent results showed that SspBCD can also constitute a defense barrier with products encoded by a three-gene cluster *sspFGH* to provide protection against phage infection in a mechanism different from that of SspBCD-SspE^59^. Interestingly, SspBCD can simultaneously couple with both SspFGH and SspE to confer an additive level of phage resistance compared to that for either barrier alone^59^. These features render the PT-based Ssp defense barrier an effective genetic module in developing phage-resistant *E. coli* host strains. Moreover, both SspBCD-SspE and SspBCD-SspFGH modules have been observed in phylogenetically diverse bacterial strains, which points to the potential application of the Ssp defense in a broader range of laboratory and industrial strains. Although all the key *E. coli* strains (K-12, B, and W) have been examined in this study, we cannot claim that the defense system developed here will protect all the other *E. coli* strains having different genetic background. For those *E. coli* strains not frequently employed, each strain must be tested or adjusted before applications. The extension of our strategy in bacterial species other than *E. coli* is somewhat limited by the knowledge gap on phage receptors and host factors important in infection pathways. Two recent papers on *E. coli* phage resistance characterization present different systematic exploration technologies to rapidly and comprehensively identify genetic determinants important in host-bacteriophage interactions^65,66^. This will facilitate the expansion of gene target sites that can be used to develop phage-resistant industrial strains.

Before phage-resistant strains can be used for industrial applications, its stability in serial passages and what frequency mutant phages evolve to escape the defense of Ssp system need to be understood. We examined the stability of the engineered *E. coli*-EPR strain by monitoring the Ssp-mediated antiphage activity every 12 h growth and 47 transfers (corresponding to 400 generations). It was found that the *E. coli*-EPR strain still exhibited the same phage resistance even after 400 generations (Supplementary Fig. 8). Furthermore, as shown in Fig. 4e–g, we performed 48-hour fermentations of three EPR *E. coli* strains challenged with phage cocktail. If phages can evolve to evade the phosphorothioation-based Ssp defense module or employ secondary receptors, the fermentation would have collapsed at a later fermentation phase. However, we did not observe the emergence of phages that can overcome host defenses even after 48 h of fermentation. These results of stability during the transfers for 400 generations and 48-hour fermentations suggest that phages cannot easily evolve and evade the defense system developed in this study. Also, the results showing that *E. coli*-EPR maintained the efficient production of target proteins in serial passages indicate that the fermentative capabilities had not been compromised in any way during the acquisition of Ssp-based phage resistance. Also, the Ssp defense system imposes no significant metabolic burden on the host *E. coli* cells, highlighting the potential use of the engineered strains in the industry.

Although not likely, the potential risks associated with accidental releases of EPR *E. coli* cells into the environment and uncontrolled horizontal gene transfer processes can be mitigated by further engineering if needed. An effective strategy is to equip the EPR *E. coli* strains with kill switch systems which can limit survival of microorganisms using a variety of mechanisms, e.g., expression of lysis or toxic proteins, cleavage of bacterial genomes and degradation of essential proteins, etc^67–69^. Kill switches can be designed to induce cell death in response to supplied inducers or external environmental signals, e.g., chemicals, temperature, pH and synthetic amino acids. Controllable kill switches have been applied in *E. coli* strains with highly stability^68^.

In summary, we report the development of phage-resistant *E. coli* strains by the use of the SspBCDE defense system. These engineered strains showed up to 10^6^-fold protection against a wide range of phages. From the perspective of industrial development, our work is of great importance because a 3-step feasible strategy was developed for engineering industrial *E. coli* strains that can be protected against phage infection. Furthermore, the strategy of simultaneous genomic integration of the *sspBCDE* module and mutations of components essential for the phage life cycle allowed the development of EPR *E. coli* strains showing strong resistance to various phages, while maintaining growth and recombinant protein production comparable to the parent strains in both flask and fed-batch cultures. The defense systems that confer resistance to phage are chromosomally integrated and encoded, and thus confer stable phage resistance phenotype and obviate selective pressure required for the plasmid-borne systems. These desirable features of stable phage resistance have been much awaited in developing industrial strains. It is believed that the PT-based antiphage system developed here will be useful for developing phage-tolerant industrial *E. coli* strains for the production of recombinant proteins and other products, including chemicals and materials.

## Methods

### Bacterial strains, bacteriophages, and media

All the strains, plasmids and bacteriophages used in this study are listed in Supplementary Data 2 and Supplementary Table 2. The primers used are listed in Supplementary Data 3. LB medium (10 g/L tryptone, 5 g/L yeast extract and 10 g/L NaCl) and LB solid agar plates (5 g/L yeast extract, 10 g/L tryptone, 10 g/L NaCl and 15 g/L agar) were used to culture all the *E. coli* strains. An appropriate concentration of antibiotic (50 µg/mL for kanamycin (Kan) and/or 36 µg/mL for chloramphenicol (Cm) and/or 100 µg/mL for ampicillin (Amp)) was added to the medium when necessary. PrimeSTAR Max DNA Polymerase (Takara, Japan) was used for polymerase chain reaction (PCR) according to standard procedures. *E. coli* DH10B was used as the host strain to perform plasmid cloning and maintenance.

### Plasmid construction

The *sspBCDE* gene cluster was cloned from *E. coli* 3234/A to construct the recombinant plasmid pWHU3640. Genomic DNA of *E. coli* 3234/A was isolated using a bacterial DNA kit (OMEGA Bio-Tek, USA) and served as a template to amplify the ~8 kb *sspBCDE* fragment using the primer pair 3640-sspBCDE-F/3640-sspBCDE-R by PCR (Supplementary Data 3). The amplified fragment was sequenced and inserted into a BamHI- and HindIII-digested pBluescript II SK(+) plasmid using a Gibson assembly cloning kit (Yeasen, China). Then, the products assembled from these two fragments were transformed into component *E. coli* DH10B cells. For the construction of plasmid pWHU6404, the gene encoding nsp8 was synthesized by TsingKe (Wuhan, China), and the resultant DNA product was ligated into pET-28a(+) that had been treated with NdeI and XhoI. Then, the ligated products were amplified in *E. coli* DH10B cells.

### Construction of engineered phage-resistant *E. coli* strains

Genome integration of the *sspBCD* gene cluster: First, the upstream (600 bp) and downstream (526 bp) fragments flanking SS9 and the 5.4 kb *sspBCD* gene cluster were amplified using the primer pairs sspBCD-KI-F1/sspBCD-KI-R1, sspBCD-KI-F3/sspBCD-KI-R3 and sspBCD-KI-F2/sspBCD-KI-R2, respectively (Supplementary Data 3). These three fragments were further fused into a single 6.6 kb fragment by overlap extension PCR. Then, this fragment was sequenced and inserted into a BamHI- and SalI-digested pKOV-Kan plasmid, generating pWHU6401. Finally, the pWHU6401 plasmid was transformed into *E. coli* MG1655, W3110 and MG1655 (DE3) to generate *E. coli* int(*aslA*-*glmZ*)::*sspBCD* by two-step double crossover.

Then, lambda red recombineering was employed to further integrate the *sspE* gene^70^. First, the 807 bp left arm chromosomal region and 2.6 kb *sspE* gene were simultaneously amplified using the template plasmid pWHU3640 and primer pair sspE-KI-F1/sspE-KI-R1 (Supplementary Data 3). Then, the 790 bp right homologous arm was amplified using the primers sspE-KI-F3/sspE-KI-R3, and the 1.3 kb FLP recognition target (FRT) sites flanking the kanamycin resistance gene were amplified using the template plasmid pKD13 and primer pair sspE-KI-F2/sspE-KI-R2. These three fragments were further fused into a single 5.5 kb fragment by overlap extension PCR. Then, the combined PCR product was transformed into *E. coli* int(*aslA*-*glmZ*)::*sspBCD* cells already expressing lambda red recombinase from the red helper plasmid pKD46 by induction with 0.2% arabinose. Successful recombination colonies were verified from the colonies grown in LB plates containing kanamycin by colony PCR. The FLP recombinase-expressing plasmid pCP20 was used to eliminate the kanamycin marker gene from the manipulated genome. All the plasmids involved in genome manipulation were cured from cells by overnight incubation at 42 °C. The successful genome integration mutants were further confirmed by genome sequencing and named *E. coli*-PT.

Construction of *fhuA*(Δ552–558 NSEG): First, the left homologous arm (709 bp) and right homologous arm (711 bp) were amplified using the primer pairs fhuA-KO-F1/fhuA-KO-R1 and fhuA-KO-F2/fhuA-KO-R2, respectively (Supplementary Data 3). The peptide NESG was inserted into the deletion region to prevent structural restrictions in β-barrel formation. Then, these two fragments were fused into a 1.5 kb single fragment by overlap extension PCR. This fused fragment was sequenced and inserted into a BamHI- and SalI-digested pKOV-Kan plasmid, generating pWHU6402. Finally, the pWHU6402 plasmid was transformed into *E. coli*-PT strains to generate *E. coli*-PT *fhuA*(Δ552–558 NSEG) strains by two-step double-crossover mutation.

Construction of Δ*trxA*: First, the left homologous arm (731 bp) and right homologous arm (553 bp) were amplified using the primer pairs trxA-KO-F1/trxA-KO-R1 and trxA-KO-F2/trxA-KO-R2, respectively (Supplementary Data 3). Then, these two fragments were fused into a 1.3 kb single fragment by overlap extension PCR. This fused fragment was sequenced and inserted into a BamHI- and SalI-digested pKOV-Kan plasmid, generating pWHU6403. Finally, the pWHU6403 plasmid was transformed into *E. coli*-PT *fhuA*(Δ552–558 NSEG) strains to generate *E. coli*-EPR strains by two-step double-crossover mutation.

### Phage propagation and cultivation

One hundred microliters of overnight host bacterial culture were mixed with 50 µL of phage stock solution, and then 4 mL of prewarmed semisolid overlay agar (LB broth with 0.6% agar) was added to spread the mixture on LB agar plates. After overnight incubation at 37 °C, 3–5 mL of SM preservative fluid (2 g/L MgSO_4_·7H_2_O, 5.8 g/L NaCl, 50 mL 1 M Tris-HCl, pH 7.4) was added to the plates. Then, the supernatant solution was collected and filtered through a 0.22 µm filter, and the fresh phage stock solutions were stored at 4 °C. The lambda phage (purchased from New England Biolabs) used in this study carries a mutation in the *cI* gene encoding a phage repressor, which ensures a default lytic pathway at the temperatures of 37 °C and above, and switches to a lysogenic pathway when temperature drops to e.g. 30 °C^71^ (Supplementary Table 2). In our experiments, the cultivation temperature of both host strains and lambda phage was at 37 °C, and thus phage lambda undergoes the lytic pathway. As shown by Gordeeva et al.^72^, lambda *cI*_*857*_ phage is capable of infecting *E. coli* cells even in the absence of induction.

### Phage infection growth curves

Bacterial strains that were inoculated overnight at 37 °C were diluted 1:100 into 10 mL of LB medium. Phages were added at different MOIs when the OD_600_ value of the cultures reached 0.6, and then 200 µL volumes of the mixture were dispensed into 96-well plates to monitor the growth of each culture by a microplate spectrophotometer (Multiskan GO SkanIt Software 6.0 version, Thermo Fischer Scientific) at 37 °C with agitation for 12 h. The OD_600_ was measured every 30 min until the end of the experiment. LB was used as a reference, and a final concentration of 100 µg/mL ampicillin was added to the medium to maintain the control empty vector and plasmid pWHU3640.

For shake flask culture, bacterial strains that were inoculated overnight at 37 °C were diluted 100-fold (v/v) into 50 mL of LB medium that was contained in 250 mL flasks. Phages were added at different MOIs when the OD_600_ value of the cultures reached 0.6 and then inoculated at 37 °C with agitation for 14 h. The growth of each culture was monitored by measuring the OD_600_ by a spectrophotometer (Ultrospec 3000, Pharmacia, Sweden) every 2 h. Samples were diluted to keep the absorbance values below 0.6 and LB was used as a reference.

### Plaque assays

Five hundred microliters of bacterial culture (OD_600_ of 0.6–0.8) was mixed with 10 mL of prewarmed semisolid overlay agar and then immediately poured onto the prepared LB agar plates. Three microliter volumes of tenfold serially diluted fresh coliphage solutions were spotted onto the bacterial lawn, and then the plates were placed in an upright position and incubated at 37 °C for 12 h.

To evaluate the EOP, 200 µL of bacterial culture (OD_600_ of 0.6–0.8) was mixed with 100 µL of serially diluted phage solution. Then, 4–5 mL of melted semisolid overlay was added and quickly poured onto a solid LB agar plate. After overnight incubation at 37 °C, plaques on the plates were counted to calculate the EOP values^73^.

### RT–qPCR assay

To extract the total RNA, 5 mL *E. coli* strain was collected and washed twice with PBS, and then 1 mL TRIzol reagent (Sigma**–**Aldrich) was added to achieve RNA extraction according to the manufacturer’s protocol. Then, reverse transcription and quantitative PCR were performed, and the corresponding qPCR primers are summarized in Supplementary Data 3.

### Cultivation conditions

To produce DAAO, transformants from kanamycin/LB plates were first inoculated in LB medium overnight at 37 °C to prepare seed cultures. Seed cultures were diluted 1:100 and grown in LB medium at 37 °C for 6–8 h. Then, 40 mL cell cultures (OD_600_ value of 2.0) were added into a 2 L baffle flask containing 1 L of LB medium and inoculated at 37 °C. The phage cocktail at a final concentration of 4.0 × 10^9^ PFU/L each of T4 and T7, 2.0 × 10^10^ PFU/L of lambda and 4.0×10^11^ PFU/L each of T1, JMPW2, T5, and EEP was added to the baffle flask at the start of cultivation. The same volume of SM buffer was added as the phage cocktail blank control, and antibiotics were added to the culture medium when appropriate.

To produce the coronavirus encoding nonstructural protein nsp8, transformants from kanamycin/LB plates were first inoculated overnight at 37 °C to prepare seed cultures. Seed cultures were diluted 1:100 and grown in LB medium at 37 °C for 6–8 h. Then, 40 mL cell cultures (OD_600_ value of 2.0) were added into a 2 L baffle flask containing 1 L of LB medium and inoculated at 37 °C. The phage cocktail at a final concentration of 4.0 × 10^9^ PFU/L each of T4 and T7, 2.0 × 10^10^ PFU/L of lambda and 4.0×10^11^ PFU/L each of T1, JMPW2, T5, and EEP was added to the baffle flask at the start of cultivation. When the OD_600_ reached 0.6, 0.1 mM IPTG was added, and then the temperature was adjusted to 28 °C. The solution was cultivated until 18 h. The same volume of SM buffer was added to set as the phage cocktail blank control, and antibiotics were added to the culture medium when appropriate.

Fed-batch fermentations were performed in a 5 L bioreactor (BIOTECH2002, Shanghai Baoxing, China) with dissolved oxygen (DO), temperature, agitation speed and pH controlled. Transformants from kanamycin/LB plates were first inoculated at 37 °C overnight to prepare seed cultures. Seed cultures were diluted 1:100 and grown in LB medium at 37 °C for 6–8 h. Then, 100 mL of cell cultures (OD_600_ value of 2.0) was inoculated into a fermenter containing 1.9 L of culture medium containing 20 g/L tryptone, 14 g/L yeast extract, 10 g/L NaCl and 20 g/L glycerol. The pH was automatically maintained at 7.5 with the addition of 5 M NaOH. Airflow was set at a rate of 4 L/min, and the agitation speed was set at 400–600 rpm. The feeding solution contained 200 g/L glycerol, 40 g/L tryptone and 20 g/L yeast extract. Antifoam 204 (Sigma–Aldrich) was automatically added into the fermenter to repress foam formation. When appropriate, antibiotics were added to the culture media as described above. The phage cocktail at a final concentration of 5.0 × 10^9^ PFU/L each of T4 and T7, 2.5 × 10^10^ PFU/L of lambda and 5.0 × 10^11^ PFU/L each of T1, JMPW2, T5, and EEP was added to the culture medium at the start of fermentation. For the fermentation of DAAO, the temperature was maintained at 37 °C, and complete fermentation lasted 48 h. For fermentation to produce nsp8 protein, seed cultures were cultivated at 37 °C, and when the OD_600_ reached 20, 1 mM IPTG was added to induce protein expression. The cultivation temperature was adjusted to 28 °C until the end of the experiment. The complete fermentation lasted 48 h.

### Analytical procedure

The DAAO activity was calculated by measuring the pyruvic acid produced by the oxidation of D-alanine^74^. The enzyme activity assay was performed as follows: 2 mL of cells was collected and then washed and resuspended in 1 mL of 75 mM disodium pyrophosphate buffer (pH 8.5). Sonication was employed to disrupt the cell walls, and centrifugation was subsequently employed to remove cell debris (16,060 × g, 10 min). Two hundred microliters of crude extract and 100 µL of 100 mM D-alanine solution were mixed and then incubated at 25 °C for 10 min. Then, 150 µL of 2 mM 2,4-dinitrophenylhydrazine (DNP) solution was added to the mixture to generate the corresponding DNP derivatives. The mixture was then incubated at 37 °C for 10 min. Finally, 1.05 mL of 0.6 N NaOH was added to the mixture to end the reaction, and the absorbance of the reaction mixture at 445 nm was measured to determine the concentration of pyruvic acid. A negative control group was prepared using the same procedure, but DAAO was replaced by 75 mM disodium pyrophosphate buffer. A calibration curve was generated by recording the absorbance values of serial dilutions of pyruvic acid (from 20 to 500 µM) with DNP under the same experimental conditions. The amount of enzyme required to convert 1 μmol of D-alanine into pyruvic acid per minute at 25 °C was defined as one unit of enzyme activity^45^.

Expression of the recombinant nsp8 protein was analyzed using SDS–PAGE (15%) with a 4% stacking gel. Five milliliters of cells were harvested, washed, and resuspended in 1 mL of PBS buffer. Sonication was employed to disrupt the cell walls, and centrifugation was subsequently employed to remove cell debris (16,060 × g, 10 min). After SDS–PAGE loading buffer was added and mixed with the supernatant, the mixture was denatured in a 95 °C dry bath for 10 min and then loaded into SDS–PAGE lanes. A 14-120 kDa Blue Plus^TM^ protein marker (TransGen, Wuhan, China) was used as a reference. The SDS–PAGE gels were stained with Coomassie Brilliant Blue G-250 (Sigma–Aldrich, St. Louis, MO), and the protein concentrations were determined by a Bradford protein assay kit (Beyotime, Wuhan, China) with bovine serum albumin (BSA) as the standard.

### Strain stability assays

To determine phage resistance inheritance and fermentation stability, three EPR-*E. coli* strains were cultured at 37 °C and transferred every 12 h at a dilution of 1:1000. The experiments were conducted for approximately 400 generations by 47 transfers^75^. Then, the strains of every 20 generations were set as experimental groups to check fermentation stability by shake flask culture, and the strains of every 40 generations were set as experimental groups to check phage resistance by phage plaque assays.

### Reporting summary

Further information on research design is available in the Nature Research Reporting Summary linked to this article.

## Supplementary information

Supplementary Information

Description of Additional Supplementary Files

Supplementary Data 1

Supplementary Data 2

Supplementary Data 3

Reporting Summary

## Data Availability

The authors declare that all data supporting the findings of this study are available within the paper and its supplementary information files. A reporting summary for this article is available as a Supplementary Information file. Source data are provided with this paper.

## Source data

Source Data
