# Supplementary material for: Systematic strategies for developing phage resistant Escherichia coli strains
Source: Nat Commun. 2022 Aug 2;13:4491. doi: 10.1038/s41467-022-31934-9 (PMC9345386; doi:10.1038/s41467-022-31934-9)
Supplement: Supplementary file 1 — Supplementary Information [file 41467_2022_31934_MOESM1_ESM.pdf]

# Systematic strategies for developing phage resistant *Escherichia coli* strains

Zou *et al.*

**Supplementary Table 1. Real-time quantitative PCR (RT–qPCR) results of the relative expression ratio of *sspE* and *sspBCD*.**

| | Average $C_T$ ± SD | | | | |
|---|---|---|---|---|---|
| Cells | *sspE* | *sspBCD* | $\Delta C_T$ ± SD | $\Delta\Delta C_T$ ± SD | *sspE* fold change relative to *sspBCD* 2^ (-$\Delta\Delta C_T$ ± SD) |
| MG1655 (pWHU3640) | 19.29 ± 0.30 | 19.67 ± 0.37 | -0.38 ± 0.08 | 0 ± 0.08 | 1 (0.94-1.07) |
| MG1655-PT | 20.99 ± 0.23 | 21.62 ± 0.30 | -0.63 ± 0.12 | -0.25 ± 0.12 | 1.19 (1.06-1.29) |

Source data are provided as a Source Data file.

**Supplementary Table 2. Bacteriophages used in this study.**

| Bacteriophages | Characteristics | Sources or reference | Sequence accession number on NCBI |
|---|---|---|---|
| T4 | *Myoviridae*, lytic, dsDNA | [1] | NC_000866.4 |
| T1 | *Siphoviridae*, lytic, dsDNA | [2] | NC_005833.1 |
| JMPW2 | *Siphoviridae*, lytic, dsDNA | [3] | NC_041873.1 |
| T5 | *Siphoviridae*, lytic, dsDNA | [4] | AY543070.1 |
| T7 | *Podoviridae*, lytic, dsDNA | [5] | NC_001604.1 |
| EEP | *Siphoviridae*, lytic, dsDNA | [6] | NC_012223.2 |
| lambda | *Siphoviridae*, lytic or lysogenic, dsDNA (Genotype: λ $cI_{857}$ *ind 1 Sam* 7) | New England Biolabs | Based on NC_001416.1 by applying mutations: 37589 C->T, 45352 G->A, 37742 C->T and 43082 G->A |

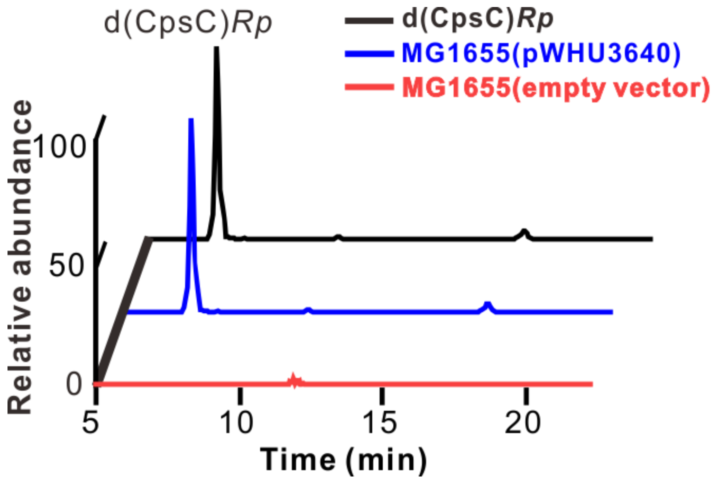


**Supplementary Fig. 1. The occurrence of DNA phosphorothioation (PT) modification at d($C_{PS}C$) in *E. coli* SspBCDE-expressing MG1655 (pWHU3640) was verified by liquid chromatography-tandem mass spectrometry (LC–MS/MS).** No DNA PT modification was detected in MG1655 cells with the empty vector pBluescript II SK(+). Chemically synthesized PT-linked d($C_{PS}C$) in the $R_P$ configuration was used as a reference. The treatment of DNA samples and LC–MS/MS parameters were used as previously described [7]. Source data are provided as a Source Data file.

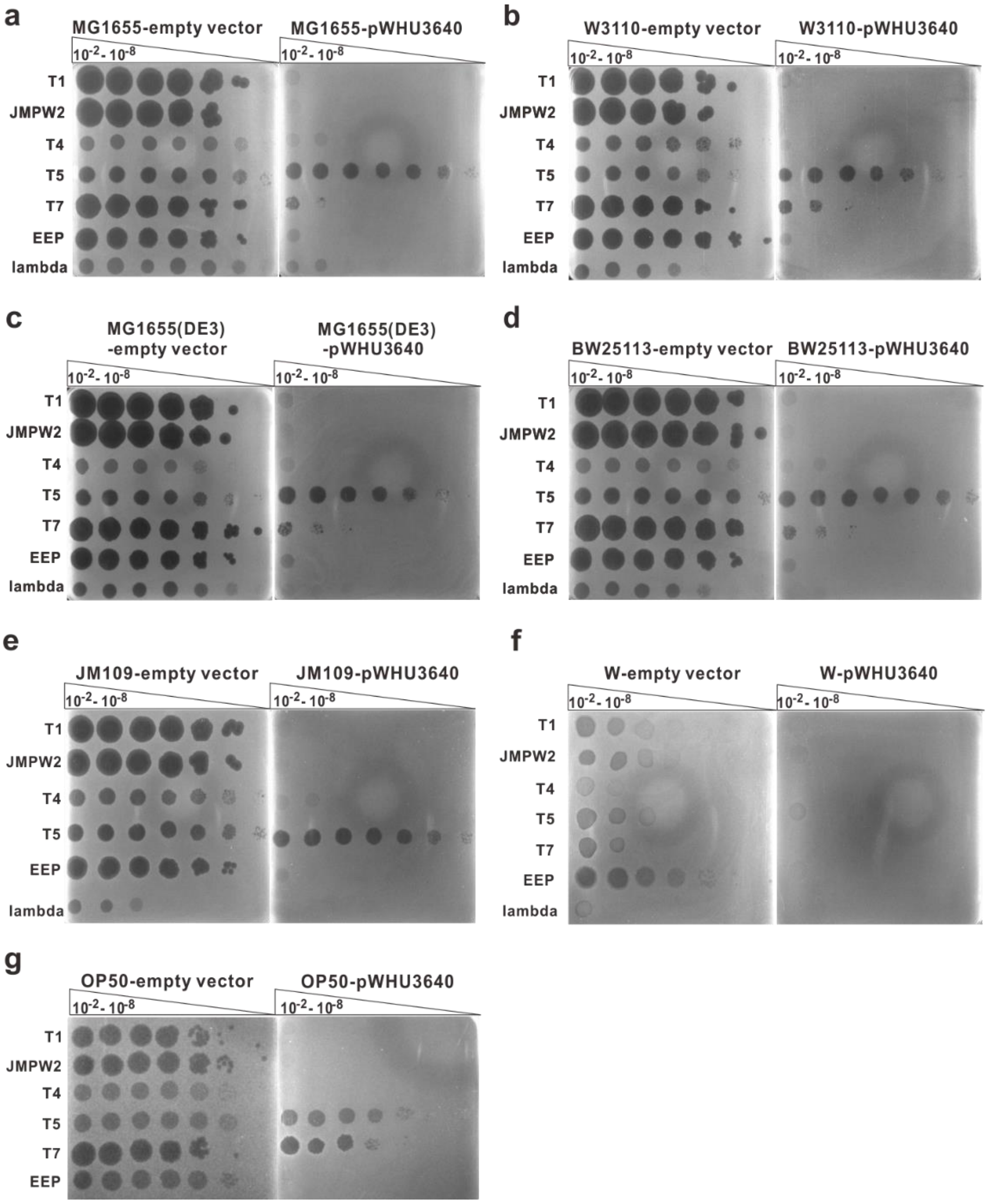


**Supplementary Fig. 2. The SspBCDE defense system provides broad protection against phage infection in different *E. coli* strains.** a-g The SspBCDE defense system was cloned into different *E. coli* hosts: MG1655 (a), W3110 (b), MG1655 (DE3) (c), BW25113 (d), JM109 (e), W (f), and OP50 (g). Phage plaque assays were used to compare the phage infection efficiencies of *E. coli* strains carrying the empty vector and *E. coli* strains carrying pWHU3640. All results were obtained at least three times. Source data are provided as a Source Data file.

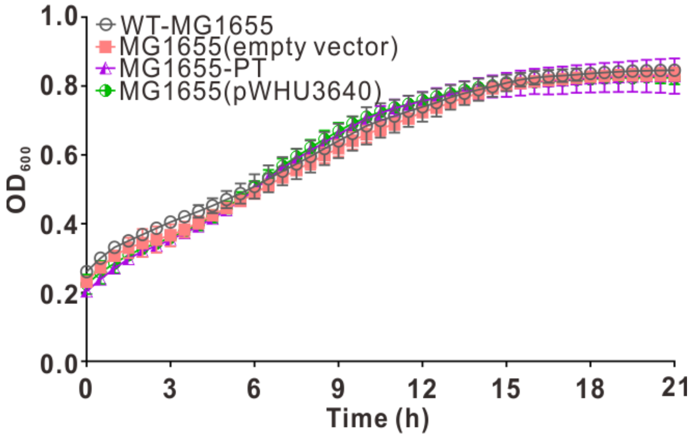


**Supplementary Fig. 3. Growth curves of the MG1655 (pWHU3640) and MG1655-PT strains.** WT-MG1655 and MG1655 (empty vector) strains were used as control groups. Data are shown as the mean ± SD of three independent experiments. Source data are provided as a Source Data file.

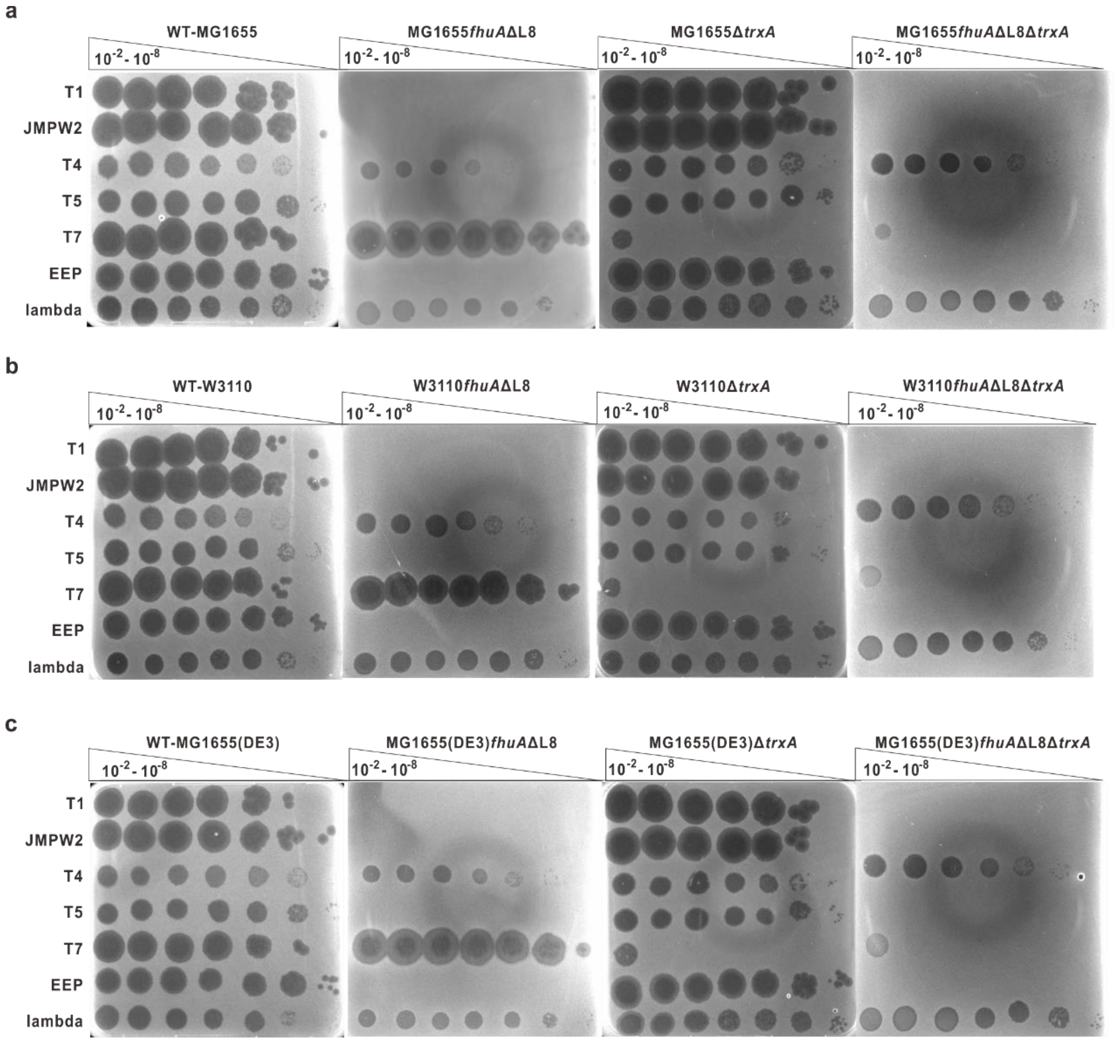


**Supplementary Fig. 4. Construction of engineered phage-resistant (EPR) *E. coli* strains by modifying genes that are essential for T5 and T7 phage infection.** Phage plaque assays were used to compare the phage infection efficiencies in WT-MG1655, MG1655*fhuA*ΔL8, MG1655Δ*trxA*, and MG1655*fhuA*ΔL8Δ*trxA* strains (a); in WT-W3110, W3110*fhuA*ΔL8, W3110Δ*trxA*, and W3110*fhuA*ΔL8Δ*trxA* strains (b)**;** and in WT-MG1655 (DE3), MG1655 (DE3)*fhuA*ΔL8, MG1655 (DE3)Δ*trxA*, and MG1655 (DE3)*fhuA*ΔL8Δ*trxA* strains (c). The results are representative of three independent experiments. Source data are provided as a Source Data file.

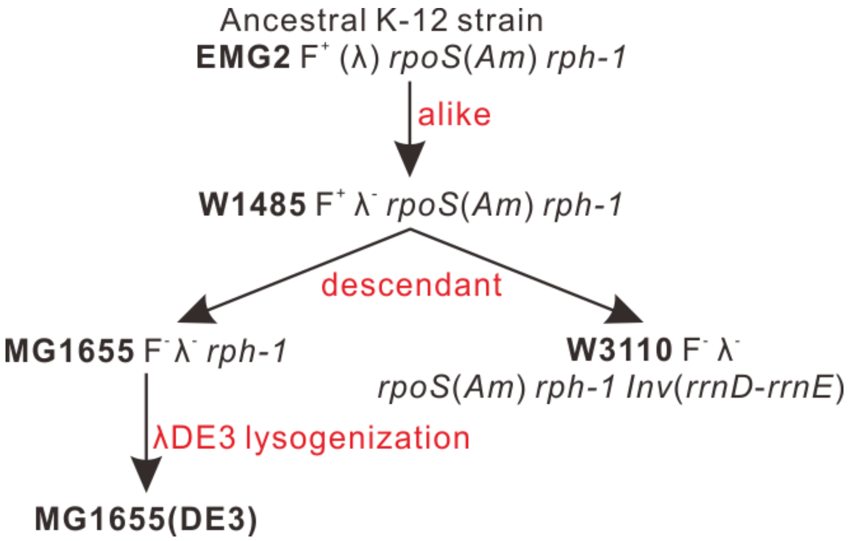


**Supplementary Fig. 5. Genetic pedigree of three *E. coli* K-12 strains, MG1655, W3110 and MG1655 (DE3).**

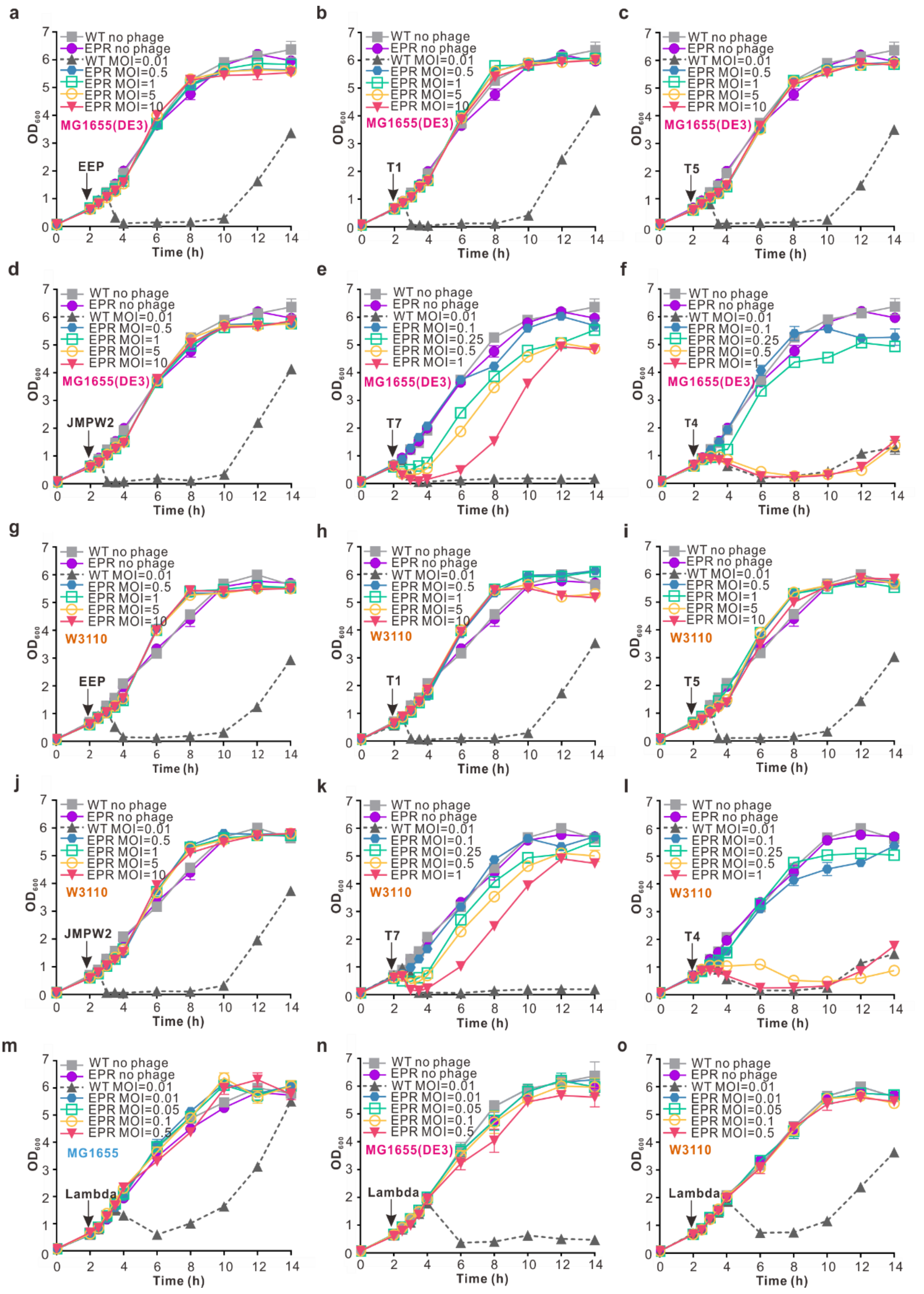


**Supplementary Fig. 6. Growth curves of W3110-EPR strains and MG1655 (DE3)-EPR strains treated with coliphages at different multiplicities of infection (MOIs).** a-f MG1655 (DE3)-EPR strains were treated with the coliphages EEP, T1, T5, JMPW2, T7, and T4 at the indicated MOIs. g-l W3110-EPR strains were treated

with the coliphages EEP, T1, T5, JMPW2, T7, and T4 at the indicated MOIs. m-o Three *E. coli*-EPR strains were treated with coliphage lambda at the indicated MOIs. Experiments were repeated three times, and each point represents the mean ± SD. Source data are provided as a Source Data file.

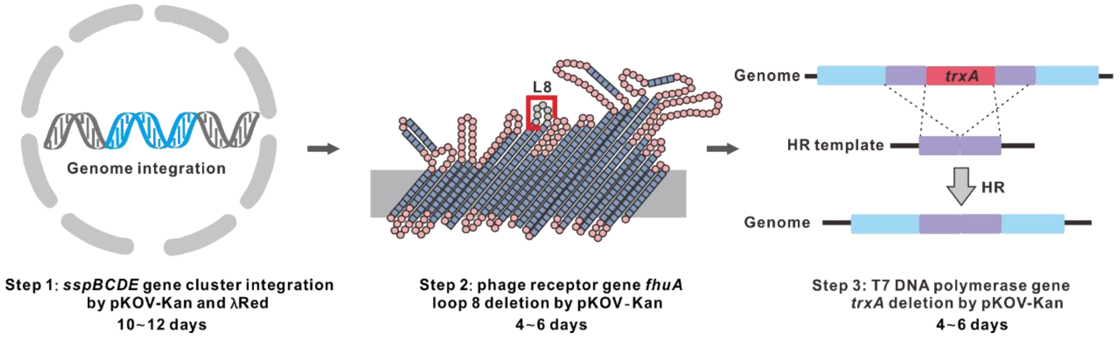


**Supplementary Fig. 7. Flow diagram of the construction of *E. coli*-EPR strains by genome integration of the *sspBCDE* gene cassette and mutations of components that are essential to phage life cycles.** HR, homologous recombination.

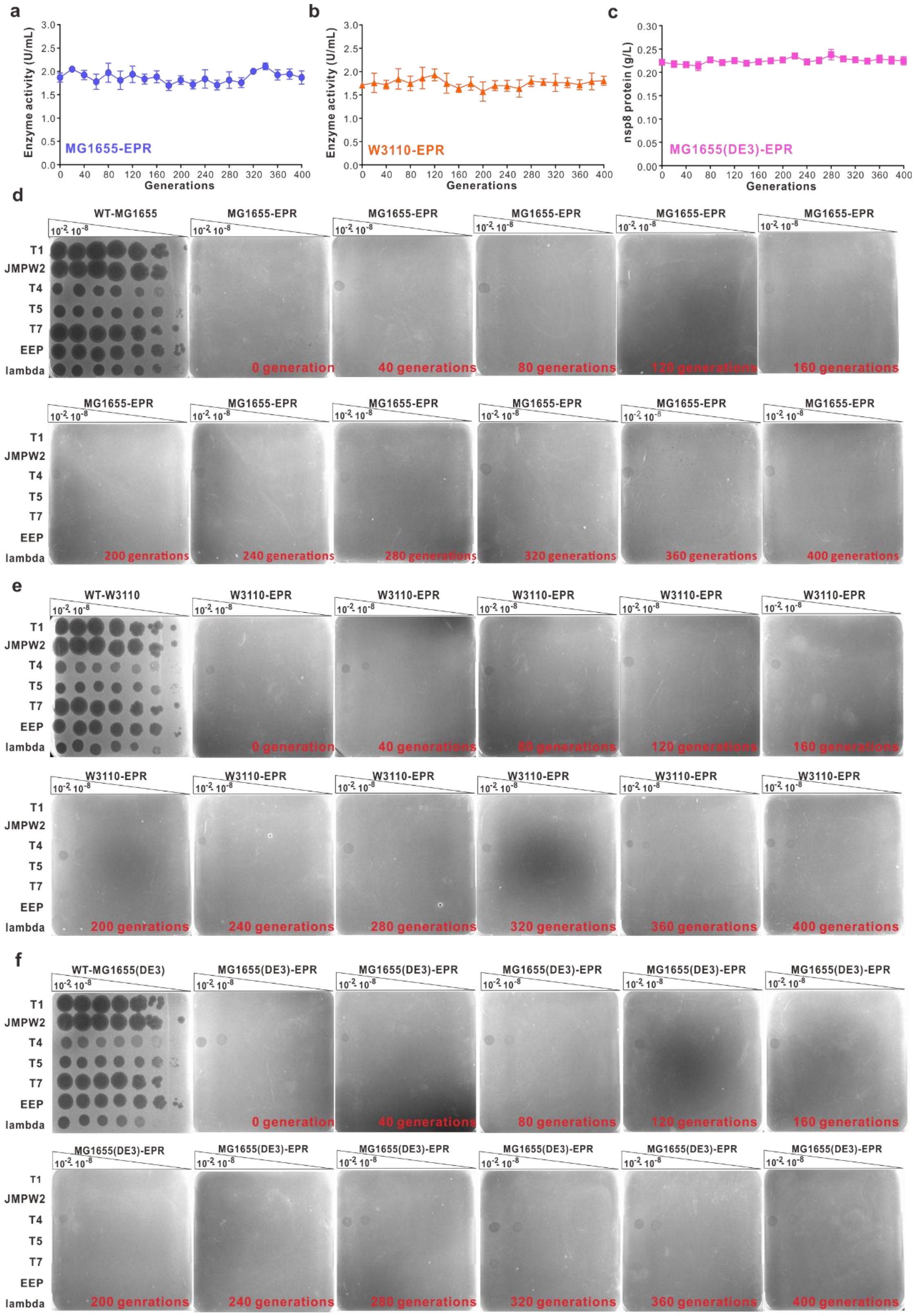


**Supplementary Fig. 8. Phage resistance inheritance and ferment stability of EPR *E. coli* strains.** Shake flask culture assays were employed to check the target protein production in the MG1655-EPR (a), W3110-EPR (b) and MG1655 (DE3)-EPR (c) strains. Data are shown as the mean ± SD of three independent experiments. And

phage plaque assays were used to compare the phage infection efficiencies in the MG1655-EPR (d), W3110-EPR (e) and MG1655 (DE3)-EPR (f) strains during the evolution experiment at 37 °C. The results are representative of three independent experiments. The experiment was conducted for 47 transfers to obtain approximately 400 generations. Then, the strains of every 20 generations were set as experimental groups to check the target protein production by shake flask culture, and every 40 generations were set as experimental groups to check phage resistance by phage plaque assays. Source data are provided as a Source Data file.

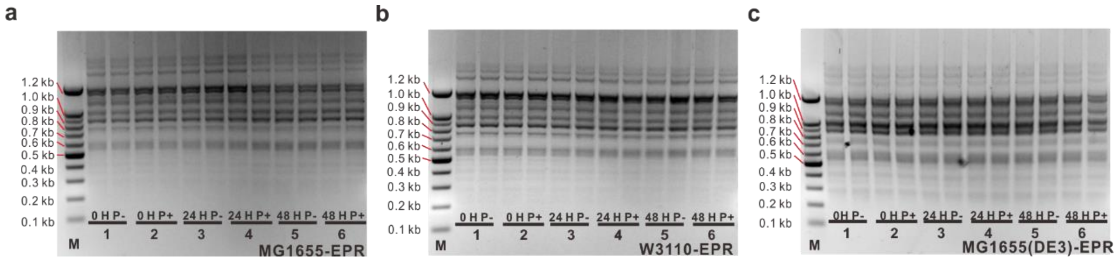


**Supplementary Fig. 9. Ligation-mediated PCR (LM-PCR) of EPR *E. coli* strains used for detection of changes in the host genome.** LM-PCR products of three *E. coli* K-12 strains, MG1655-EPR (a), W3110-EPR (b) and MG1655 (DE3)-EPR (c), were obtained from two independent experiments and loaded into adjacent wells. The sizes of the 100 bp DNA molecular weight marker are shown in lane M; lane 1, fed-bath fermentation sample at 0 h; lane 2, phage cocktail added fed-bath fermentation sample at 0 h; lane 3, fed-bath fermentation sample at 24 h; lane 4, phage cocktail added fed-bath fermentation sample at 24 h; lane 5, fed-bath fermentation sample at 48 h; lane 6, phage cocktail added fed-bath fermentation sample at 48 h. Source data are provided as a Source Data file.

## Supplementary references


1. Miller ES, Kutter E, Mosig G, Arisaka F, Kunisawa T, Ruger W. Bacteriophage T4 genome. *Microbiol Mol Biol Rev* **67**, 86-156 (2003).
2. Roberts MD, Martin NL, Kropinski AM. The genome and proteome of coliphage T1. *Virology* **318**, 245-266 (2004).
3. Shen M, *et al.* Complete genome sequences of T1-like phages JMPW1 and JMPW2. *Genome Announc* **4**, e00601-16 (2016).
4. Wang J, *et al.* Complete genome sequence of bacteriophage T5. *Virology* **332**, 45-65 (2005).
5. Dunn JJ, Studier FW. Complete nucleotide sequence of bacteriophage T7 DNA and the locations of T7 genetic elements. *J Mol Biol* **166**, 477-535 (1983).
6. Li S, *et al.* Characterization and genome sequencing of a novel coliphage isolated from engineered *Escherichia coli*. *Intervirology* **53**, 211-220 (2010).
7. Xiong X, *et al.* SspABCD-SspE is a phosphorothioation-sensing bacterial defence system with broad anti-phage activities. *Nat Microbiol* **5**, 917-928 (2020).
