# Supplementary material for: Systematic strategies for developing phage resistant Escherichia coli strains
Source: Nat Commun. 2022 Aug 2;13:4491. doi: 10.1038/s41467-022-31934-9 (PMC9345386; doi:10.1038/s41467-022-31934-9)
Supplement: Supplementary file 2 — Description of Additional Supplementary Files [file 41467_2022_31934_MOESM2_ESM.pdf]

Description of Additional Supplementary Files

File Name: Supplementary Data 1
Description: Ssp protection against phages in this study

File Name: Supplementary Data 2
Description: Strains and plasmids used in this study

File Name: Supplementary Data 3
Description: Primers used in this study
